# Crosstalk between dihydroceramides produced by *Porphyromonas gingivalis* and host lysosomal cathepsin B in the promotion of osteoclastogenesis

**DOI:** 10.1111/jcmm.17299

**Published:** 2022-04-16

**Authors:** Carolina Duarte, Chiaki Yamada, Christopher Garcia, Juliet Akkaoui, Anny Ho, Frank Nichols, Alexandru Movila

**Affiliations:** ^1^ 2814 Department of Oral Sciences and Translational Research College of Dental Medicine Nova Southeastern University Davie Florida USA; ^2^ Department of Biomedical Sciences and Comprehensive Care Indiana University School of Dentistry Indianapolis Indiana USA; ^3^ 154756 Department of Oral Health and Diagnostic Sciences University of Connecticut School of Dental Medicine Farmington Connecticut USA; ^4^ Indiana Center for Musculoskeletal Health Indiana University School of Medicine Indianapolis Indiana USA

**Keywords:** cathepsin B, lysosomes, osteoclast, phosphoglycerol dihydroceramide, *Porphyromonas gingivalis*

## Abstract

Emerging studies indicate that intracellular eukaryotic ceramide species directly activate cathepsin B (CatB), a lysosomal‐cysteine‐protease, in the cytoplasm of osteoclast precursors (OCPs) leading to elevated RANKL‐mediated osteoclastogenesis and inflammatory osteolysis. However, the possible impact of CatB on osteoclastogenesis elevated by non‐eukaryotic ceramides is largely unknown. It was reported that a novel class of phosphoglycerol dihydroceramide (PGDHC), produced by the key periodontal pathogen *Porphyromonas gingivalis* upregulated RANKL‐mediated osteoclastogenesis in vitro and in vivo. Therefore, the aim of this study was to evaluate a crosstalk between host CatB and non‐eukaryotic PGDHC on the promotion of osteoclastogenesis. According to a pulldown assay, high affinity between PGDHC and CatB was observed in RANKL‐stimulated RAW264.7 cells in vitro. It was also demonstrated that PGDHC promotes enzymatic activity of recombinant CatB protein ex vivo and in RANKL‐stimulated osteoclast precursors in vitro. Furthermore, no or little effect of PGDHC on the RANKL‐primed osteoclastogenesis was observed in male and female CatB‐knock out mice compared with their wild type counterparts. Altogether, these findings demonstrate that bacterial dihydroceramides produced by *P*. *gingivalis* elevate RANKL‐primed osteoclastogenesis via direct activation of intracellular CatB in OCPs.

## 1 INTRODUCTION

Bone metabolism depends on the balance between multinucleated osteoclast‐driven bone resorption and osteoblast‐mediated bone formation.^1^ Accelerated osteoclastogenesis contributes to various inflammatory osteolytic conditions including postmenopausal osteoporosis, inflammatory arthritis and periodontitis.^1^, ^2^ Although our understanding of the molecular hallmark features of pathogenic bone resorption has been significantly advanced by the discovery of osteoclastogenic proteins belonging to the tumor necrosis factor (TNF) superfamily, including the receptor activator of nuclear factor kappa‐Β ligand (RANKL) and TNF‐a cytokines,^3^, ^4^ emerging data suggest that ceramide sphingolipids serve as second messenger molecules and may also contribute to bone tissue homeostasis and inflammatory osteolysis.^5^, ^6^, ^7^

Ceramides are ubiquitous structural components of eukaryotic cell membranes and serve as precursors for other bioactive sphingolipid species, including dihydroceramides, sphingosine, sphingosine‐1‐phosphate (S1P) and ceramide‐1‐phosphate.^8^, ^9^ While ceramides are constantly produced by eukaryotic cells, only a limited number of gut and oral bacterial species represented by the genera *Bacteroides*, *Prevotella* and *Porphyromonas*, synthesize long‐carbon‐chain ceramides (>C16) termed phosphoethanolamine dihydroceramide (PEDHC) and phosphoglycerol dihydroceramide (PGDHC).^10^, ^11^, ^12^ Whereas the cell membrane of all Bacteroidetes contain PEDHC, the keystone pathogenic bacteria for periodontitis, *Porphyromonas gingivalis*, and *Tannerella forsythia*, additionally contain PGDHC sphingolipid.^11^ It has been reported that the gut bacteria‐derived PEDHC is an endogenous source of sphingolipids that impacts lipid homeostasis in the host colon and promotes the activation of invariant natural killer T (iNKT) cells in mice.^13^, ^14^ However, PEDHC and most of the host‐derived long‐chain ceramides cannot penetrate the mammalian cell membrane.^15^, ^16^, ^17^ We previously discovered that PGDHC penetrates the cell membrane of osteoclast precursors (OCPs) and leads to the significant upregulation of RANKL‐induced osteoclastogenesis by acting on the cytoplasmic non‐muscle myosin IIA heavy chain (Myh9).^18^ Furthermore, a previously published report provided evidence that temporal degradation of Myh9 in OCPs at early stages of RANKL‐primed osteoclastogenesis is exclusively regulated by cathepsin B (CatB), but not cathepsin L, leading to their fusion.^19^

CatB is a lysosomal cysteine protease that is ubiquitously expressed in mammalian cells and most active under acidic endosomal conditions.^20^ Published evidence clearly demonstrated that CatB is released into the cytosol following lysosomal membrane permeabilization (LMP) which in turn mediates a variety of homeostatic and pathogenic processes, including Alzheimer's disease, extracellular matrix degradation, cancer metastasis to bone and osteoporosis.^11^, ^21^, ^22^ Furthermore, CatB plays a critical role in the fusion of osteoclast precursors (OCPs) induced by RANKL.^19^

While it has been demonstrated that intracellular host ceramides increase LPM and directly activate CatB in the cytoplasm of myeloid cells,^23^ there is no clear evidence for the association between CatB activity and PGDHC in osteoclastogenesis and osteolysis. However, we recently demonstrated that PGDHC significantly elevates the expression of CatB in neurons, *in vitro*.^24^ Thus, these observations raise an intriguing question of whether PGDHC plays a role in CatB release into the cytosol leading to dramatically elevated RANKL‐induced osteoclastogenesis.

Our results indicated that PGDHC sphingolipid isolated from the key periodontal pathogen *P*. *gingivalis* promotes lysosomal leakage and activation of catB in the cytoplasm of RANKL‐primed OCPs. Furthermore, we also demonstrated that PGDHC promotes osteoclast differentiation and activation in a CatB‐dependent manner using male and female CatB‐*knock out* (CatB^−/−^) mice and their wild type counterparts (CatB^+/+^).

## 2 MATERIALS AND METHODS

### 2.1 Animals

Six‐week‐old male and female C57BL/6J mice (CatB^+/+^) were obtained from Jackson Laboratories (Bar Harbor, ME, USA). Cathepsin B knockout mice (CatB^−/−^) (Ctsb^tm1Jde^, MGI: J:47432) were also obtained from Jackson Laboratories and were further inbred in‐house to expand the colony. The genotypes of each new generation of the CatB knockout mice were examined by genomic PCR analysis using the following primers:

CatB‐Common‐F (5’‐ TGGGGACAGCCACACTCTAC‐3’), CatB‐Mutant‐R (5’‐CTGTTGTGCCCAGTCATAGC‐3’) and CatB‐WT‐R (5’‐ ATCACACCCCAACCAGTCTC‐3’). The animals were group housed and kept in a conventional room with a 12‐hour light‐dark cycle at a constant temperature. Experiments were performed in adult eight‐week‐old male and female mice that were randomly sorted into control or experimental groups. All experiments, involving studies performed with animals, were approved by the Institutional Animal Care and Use Committee (IACUC) committee at Nova Southeastern University (protocol # 2019.01.AM3‐A3).

### 2.2 Cells

Mouse monocyte/macrophage cell line RAW 267.4 (ATCC TIB‐71) cells were obtained from ATCC (Rockville, MD, USA). Bone marrow derived macrophages (BMDMs) were isolated from the femurs and tibias of male and female CatB^+/+^ and CatB^−/−^ mice. The mice were euthanized by carbon dioxide inhalation followed by neck dislocation, after which the femurs and tibias were dissected, and epiphyses removed. The bone marrow was flushed out of the remaining diaphyses with sterile PBS and viable mononuclear cells were recovered using Histopaque^®^‐1083 (Sigma‐Aldrich, Cat# 10831). Then, isolated mononuclear cells were constantely incubated with recombinant M‐CSF (30 ng/ml, BioLegend Cat# 576406).

### 2.3 RANKL‐induced osteoclastogenesis

To induce osteoclastogenesis, both RAW 264.7 cells and BMDMs were stimulated with 3 and 10 ng/ml, respectively, of recombinant mouse RANKL (Biolegend, Cat# 769406), in the presence or absence of 1 μg/ml and 10 ng/ml, respectively, of PGDHC isolated from *Porphyromonas gingivalis* (ATTC, strain #33277), which was provided by Dr. Frank Nichols (University of Connecticut, Mansfield, CT). For biological experiments, PGDHC was sonicated (2 s, 3 W) in phosphate‐buffered saline (PBS, Gibco, Cat# 10010049) to achieve a concentration of 1 μg/ml. In addition, specific experimental groups were treated with 1 μM of intracellular CatB inhibitor, CA‐074‐ME (Calbiochem, Cat# 205531).

### 2.4 Immunofluorescence

Immunofluorescence staining was performed on fixed RANKL‐primed BMDMs, blocked with 5% BSA and incubated overnight with anti‐Galectin‑3 mAb (R&D Systems, Cat# AF1197) and anti‐LAMP‐1 (CD107a) Antibody (Sigma Aldrich, Cat# AB2971). The cells were, then, incubated with Alexa Fluor^®^ 488 Goat anti‐Rat IgM Antibody (Biolegend, Cat# 408908), Alexa Fluor^®^ 488 Donkey Anti‐Goat IgG H&L (Abcam, Cat# ab150129) and Alexa Fluor^®^ 405 Goat Anti‐Rabbit IgG H&L (Abcam, Cat# ab175652). The nuclei were counterstained using Draq5^TM^ (Thermo Scientific, Cat#62254) staining solution, and the slides were mounted using Aqua‐Poly/Mount (Polysciences, Cat# 1860620). The images were acquired using a Zeiss LSM 800 confocal microscope at 20 and 40× magnification and analyzed using the Zen (Black edition), Zen 3.2 (Blue edition) and Image J software.

### 2.5 Assessment of lysosomal leakage

Lysosomes in RANKL‐primed primary BMDMs treated with PGDHC were evaluated using Acridine Orange (Immunochemistry Technologies, Cat# 6130) at a 1:1000 dilution and Hoechst 33342 (Immunochemistry Technologies, Cat# 639) nuclear counterstain at 0.5% v/v concentration, according to the manufacturer's recommendation. The images were acquired using a Zeiss LSM 800 confocal microscope and analyzed using the Zen (Black edition), Zen 3.2 (Blue edition) and Image J software.

### 2.6 Assessment of cathepsin B activity in vitro

The intracellular activation of CatB in RANKL‐primed primary BMDMs treated with PGDHC were evaluated using the Magic Red^®^ Cathepsin B substrate (Immunochemistry Technologies, Cat# 6133) and Hoechst 33342 (Immunochemistry Technologies, Cat# 639) nuclear counterstain at 0.5% v/v concentration, following the manufacturer's recommended protocol. For the *ex vivo* assessment of CatB activation by ceramides, including PGDHC, the Magic Red substrate was incubated at 37 degrees Celsius at pH 6.0 for 35 min with 100 ng/ml of mouse recombinant Cathepsin B (R&D Systems, Cat# 965‐CY) with or without 1 µg/ml of PGDHC, C16 Ceramide (d16:1/16:0) (Cayman Chemical, Cat# 24426) or C16 dihydro Ceramide (d18:0/16:0) (Cayman Chemical, Cat# 24369). The substrate degradation was measured every five minutes for 30 min in a FilterMax F5 Multi‐Mode Microplate Reader (Molecular Devices). The intracellular activity of CatB in RANKL‐primed RAW 264.7 cells was assessed using the InnoZyme™ Cathepsin B Activity Assay (Calbiochem, Cat# CBA001) following the manufacturer's recommended protocol, and was measured in the FilterMax F5 Multi‐Mode Microplate Reader.

### 2.7 Western Blot

RANKL‐stimulated RAW264.7 cells were lysed in CytoBuster^TM^ Protein Extraction Reagent (Millipore, Cat# 71009) supplemented with protease and phosphatase inhibitors. The lysates were denatured, separated in a Bolt™ 4–12% Bis‐Tris gel (Invitrogen, Cat# NW04120BOX) and transferred to a membrane using an iBlot™ 2 PVDF Gel Transfer Stack (Invitrogen, Cat# IB24001). The membrane was blocked with 5% Blotting Grade Blocker Non Fat Dry Milk (BioRad, Cat# 1706404XTU) and incubated overnight with Human/Mouse/Rat Galectin‑3 Antibody (R&D Systems, Cat# AF1197), Anti‐LAMP‐1 (CD107a) Antibody (Sigma Aldrich, Cat# AB2971) and GAPDH (14C10) Rabbit mAb (Cell Signaling Technology, Cat# 2118) in blocking buffer. The cells were, then, incubated with HRP‐linked secondary antibodies, and the protein bands were detected using the chemiluminescent HRP substrate SuperSignal™ West Pico PLUS (Thermo Scientific, Cat# 34579).

### 2.8 Pulldown assay

Whole cell protein lysate from RAW 264.7 cells was extracted using the Mem‐PER Plus mammalian Protein Extraction Kit (Thermo Scientific, Cat# 89842) following the manufacturer's recommendations. PGDHC was bound to ACROS Organics™ Glass Beads, 500–750 µm (Thermo Scientific, Cat# 397640250) as described by Green^25^ and the pulldown assay was performed as previously described by Kanzaki et al.^18^ Control unbound glass beads or PGDHC‐bound glass beads were mixed with whole cell protein lysate from RAW 264.7 cells and incubated at 4℃ with shaking overnight, separated using Spin‐X centrifuge tube filters (Corning, Cat# CLS8160) and washed three times with PBS. Proteins bound onto control unbound glass beads or PGDHC‐bound glass beads were eluted with 4× Bolt LDS sample buffer (Thermo Scientific, Cat# B0007) by brief boiling at 70℃ for 10 min. Subsequently, the protein concentration was measured with the Pierce BCA Protein Assay Kit (Thermo Scientific, Cat# 23227) and the proteins present in each LDS‐treated sample were separated by electrophoresis using a Bolt™ 4–12% Bis‐Tris gel (Invitrogen, Cat# NW04120BOX), followed by Western blot analysis as previously described, using the Anti‐Cathepsin B antibody [EPR21033] (Abcam, Cat# ab214428).

### 2.9 RNA extraction and quantitative real time polymerase chain reaction

The total RNA from RANKL‐stimulated RAW264.7 cells, as well as male and female CatB^+/+^ and CatB^−/−^ BMDMs, was extracted using the PureLinkTM RNA Mini Kit (Invitrogen), according to the manufacturer's instructions, and reverse transcription of 1 μg of total RNA was performed using the Verso cDNA Synthesis Kit (Thermo Scientific), following the manufacturer's recommendations. The gene expressions were measured using PowerUp^TM^ Sybr^TM^ Green Master Mix (Applied Biosystems Diagnostics), in the AriaMx Real‐time PCR System (Agilent) and quantified using the AriaMX Software Version 1.3. The data were analyzed using the relative standard curve method normalized to glyceraldehyde 3‐phosphate dehydrogenase (GAPDH) and presented as fold change.

### 2.10 Adherent cell TRAP staining

RANKL‐stimulated RAW264.7 cells and BMDMs were stained for tartrate‐resistant acid phosphatase (TRAP) using a leukocyte acid phosphatase kit (Sigma Diagnostics), after five or six days respectively. TRAP‐positive (TRAP+) cells with more than three nuclei were considered osteoclasts. TRAP+multinuclear cells were counted, and the results were expressed as the number of cells per well.^18^

### 2.11 A mouse calvarial injection model

To evaluate the effects of PGDHC on the promotion of CatB and CatK activities *in vivo*, a mouse model of calvarial injection was employed with some modifications (Kanzaki et al, 2017). Briefly, wild type male C57BL/6 mice were anesthetized with isoflurane (Patterson Veterinary, Cat# NDC14043‐704–06), after which they received a 100 μl intra‐periosteal injection of either of the following solutions: 1) PBS (Gibco, Cat# 10010049); 2) 10 μg/ml of recombinant RANKL (Biolegend, Cat#769406) in PBS; or 3) a mixture of 10 μg/ml of recombinant RANKL and 10 μg/ml of PGDHC in PBS. The injections were repeated every other day for 10 days.

The mouse calvaria from the calvaria injection experiments were carefully dissected and homogenized in Trizol (Invitrogen) followed by RNA purification using the RNeasy Micro Kit (Quiagen) and qRT‐PCR was performed as described above.

### 2.12 Tissue TRAP staining

Calvaria bone samples were fixed overnight in 10% neutral buffered formalin, were decalcified in 0.5 M EDTA (Millipore) pH 9.0 for 4 weeks at 4°C and, subsequently, dehydrated in graded alcohol and embedded in paraffin. Coronal sections at a thickness of 6‐μm were prepared for histological analysis and TRAP stained as described by Kanzaki et al.^18^ Deparaffinized slides were treated with 0.2 M Tris buffer at pH 9.0 for one hour at 37℃, followed by incubation in 0.2 M acetate +50mM 1 (+) tartaric acid buffer. Finally, the slides were incubated in TRAP solution (0.2 M acetate, 0.5 mg/ml naphtol and 1.1 mg/ml Fast Red) at 37℃ for 2 h, counterstained with haematoxylin for five seconds and mounted.

### 2.13 In vivo cathepsins activity assay

At Day 10 after mouse calvaria injections were initiated, 100 µl of a CatB™ 680 FAST or CatK™ 680 FAST fluorescent probes (Perkin Elemer, Cat# NEV11112 and Cat# NEV11000) was administrated intravenously, to each mouse. After 6 hours, the luminescence intensity was measured using the In‐Vivo Xtreme (Bruker) animal imaging system. A circular region of interest (ROI) was defined as the area which exhibited more than 50% of maximum luminescence in the inflammatory site, for each mouse. The total flux measured in photons per second in the ROI was quantified using the In‐Vivo Xtreme Software (Bruker) according to the manufacturer's instructions.

### 2.14 Statistical analysis

Differences in quantitative data were determined by One‐Way ANOVA followed by Tukey's post‐hoc test, whereas kinetic CatB activity as assessed using repeated measures ANOVA followed by Tukey's pot hoc test, using Prism 9.0.0 (Graphpad). The p value below 0.05 was considered significant. The data in the graphs are expressed as the group mean plus standard deviation.

## 3 RESULTS

### 3.1 *Porphyromonas gingivalis*‐derived PGDHC promotes lysosomal membrane permeabilization (LMP) in RANKL‐primed OCPs

**FIGURE 1:**
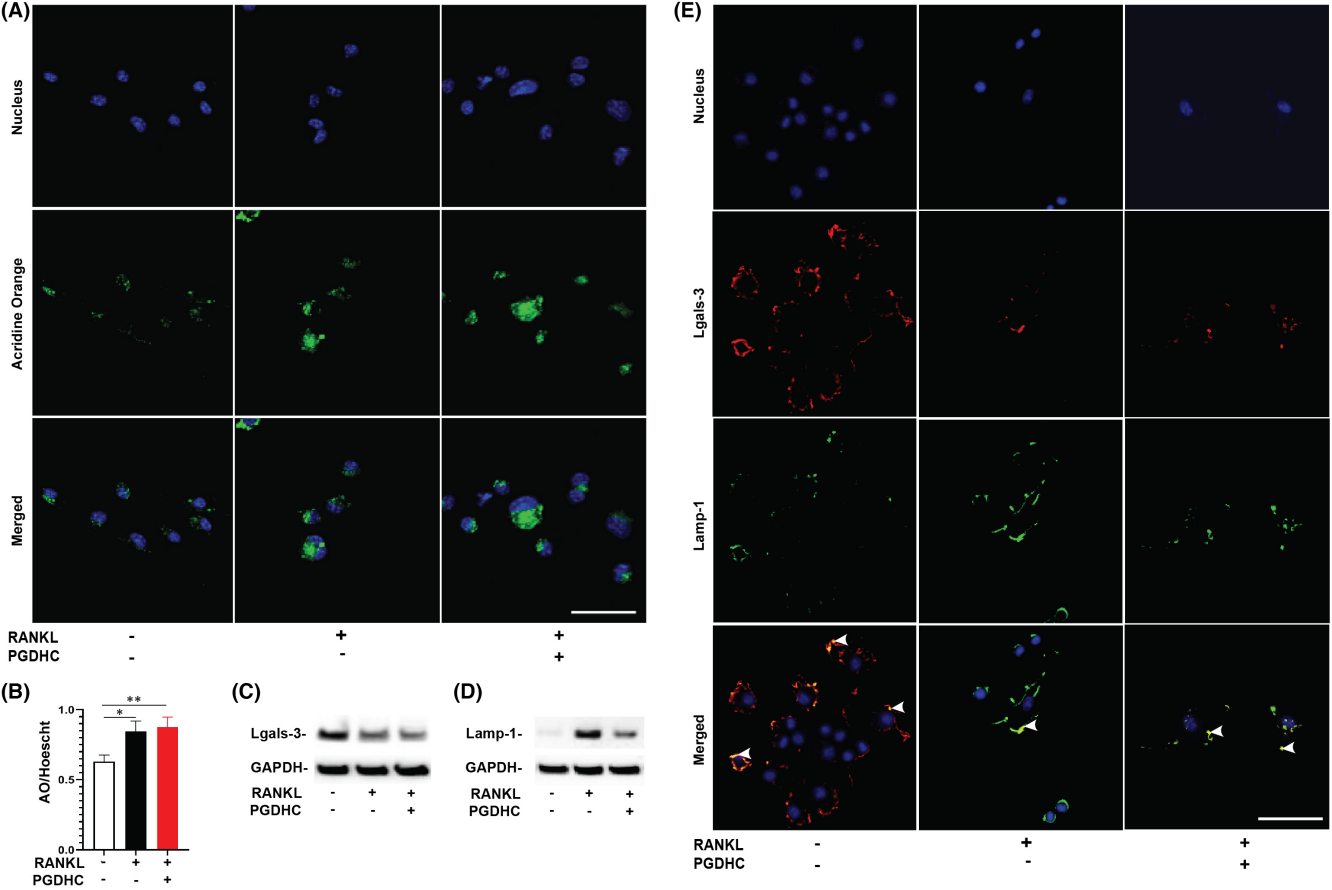
PGDHC promotes lysosomal membrane permeabilization in RANKL‐primed bone marrow‐derived macrophages (BMDMs) *in vitro*. Representative images of acridine orange staining (AO, green) and nuclei (Hoeschst; blue) (A) and fluorescence intensity quantification (B) in the cytoplasm of RANKL‐primed BMDMs after 48 h stimulation with PGDHC; fluorescence intensity was quantified as the ratio of AO/Hoechst. (C – D) Identification of galectin 3 (Lgals3) and lysosome associated membrane protein 1 (Lamp‐1) proteins associated with the lysosomal membrane permeabilization in the cell lysate of BMDMs after 48 h stimulation with RANKL and PGDHC. (E) Immunofluorescent staining of Lgals‐3 (red), Lamp‐1 (green), and nuclear counterstain (blue). Arrow heads point to areas of colocalization of Lgals‐3 and Lamp‐1. *p < 0.05; **p < 0.01; Scale bars, 50 μm

Since intracellular accumulation of ceramide species of mammalian origin causes lysosomal membrane permeabilization (LMP) followed by the relocation of lysosomal cathepsins to the cytoplasm (Puissant, Colosetti et al. 2010; McMichael, Wysolmerski et al. 2009), we first examined whether the cell permeable PGDHC promotes LMP in RANKL‐primed BMDMs, as the source of OCPs. Using the lysosomotropic acridine orange (AO) fluorescent dye,^26^, ^27^, ^28^ we found that RANKL stimulation significantly elevated the concentration and diffusion of AO in the OCPs cytosol, and PGDHC further increased AO concentration and diffusion compared with the control cells where AO aggregates were observed, indicating that *P*. *gingivalis*‐derived PGDHC elevated the loss of lysosomal membrane integrity during RANKL‐mediated osteoclastogenesis (Figure 1A,B).

Published evidence indicated that galectin‐3 (LGALS3) mediates repair of the damaged lysosomal membrane and decreases the traffic of cathepsins, including CatB, into the cytosol, while the lysosome associated membrane protein 1 (LAMP1) stabilizes the lysosomal membrane after LMP.^22^, ^29^, ^30^, ^31^ Thus, to further assess the effect of PGDHC on lysosomes in OCPs, we examined the expression patterns of LGALS3 and LAMP‐1 in RANKL‐primed BMDMs using Western blot analysis and confocal microscopy assays. In the presence of PGDHC, the levels of LGALS3 and LAMP‐1 were significantly diminished in RANKL‐stimulated BMDMs compared with the cells treated with RANKL alone (Figure 1C). In addition, the co‐localization of LGALS3 and LAMP‐1 was significantly diminished in RANKL‐primed BMDMs exposed to PGDHC (Figure 1D). Altogether these data indicate that *P*. *gingivalis*‐derived PGDHC promotes LMP and destabilizes the lysosomal membrane which in turn may promote relocation of cathepsins, including CatB, to the OCP’s cytosol.

### 3.2 *Porphyromonas gingivalis*‐derived PGDHC increases the enzymatic activity of CatB in the cytosol of mouse OCPs and mouse calvaria

**FIGURE 2:**
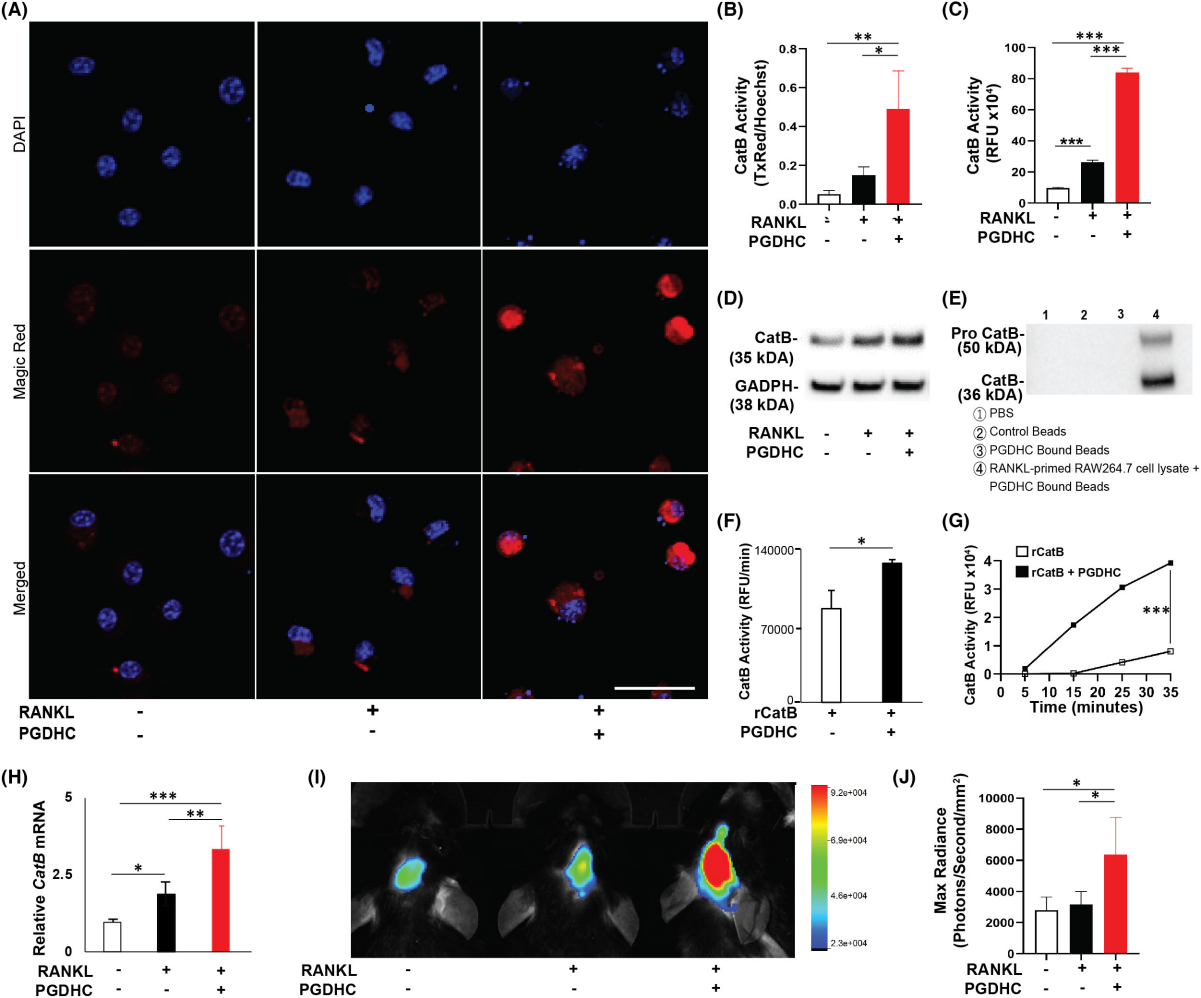
PGDHC elevates Cathepsin B (CatB) enzymatic activity in RANKL‐primed osteoclast precursors *in vitro* and in mouse calvaria *in vivo*. (A) Immunostaining of CatB activity in bone marrow derived macrophages (BMDMs) after 48 h stimulation using Magic Red showing areas of CatB substrate digestion (TxRed,red) and nuclear couterstain (Hoescht, blue). (B) Quantification of the fluorescence intensity presented as the ratio of TxRed/Hoechst. (C) CatB activity measured in the cell lysate of BMDMs after 48 h stimulation using a substrate‐based assay. (D) Identification of CatB protein in the cell lysate of BMDMs after 48 h stimulation, and (E) identification of pro‐CatB and CatB proteins from the product of a pull down assay using cell lysate of RANKL‐primed RAW 264.7 cells, by Western blot analysis. (F) CatB substrate digestion speed and (G) total digestion rate by the recombinant CatB and recombinant CatB with PGDHC, *ex vivo*. (H) The *CatB* gene expression measured using qRT‐PCR analysis from tissue lysates of wild‐type mouse calvarias injected with RANKL or a combination of RANKL‐PGDHC. (I) Live imaging and (J) quantification of CatB in wild‐type mouse calvarias injected with RANKL or a combination of RANKL‐PGDHC, using *in vivo* bioluminescence imaging (Extreme II, Bruker) with a CatB specific fluorescent probe (Pro Sense 680, Perkin Elmer). *p < 0.05; **p < 0.01; ***p < 0.001; Scale bar, 50 μm

Because our data indicate that PGDHC promotes LMP and leakage of lysosomal enzymes in OCPs (Figure 1A), we sought to elucidate whether PGDHC contributes to the activation of CatB in the cytosol of OCPs. In our live cell observations, no or little amount of CatB and activated CatB was detected in the cytosol of RANKL‐stimulated BMDMs and untreated cells, whereas a significantly increased release and activation of CatB in the cytoplasm of RANKL‐primed BMDM was detected in response to PGDHC (Figure 2A,B). Furthermore, PGDHC significantly elevated the enzymatic activity of CatB in whole cell lysate from RANKL‐stimulated BMDMs compared with the sham stimulated cells measured after 48h, when OCPs progress towards differentiation (Figure 2C). In addition, the expression of CatB protein in the cell lysate of OCPs treated with RANKL/PGDHC was significantly higher compared with the control group and the group treated with RANKL alone (Figure 2D; Figure S1). Thus, these data indicate that PGDHC ceramide promotes CatB expression and activity in the cytosol of OCPs. Furthermore, the emergent effect of PGDHC on the protein expression and enzymatic activity of CatB was not observed in the ubiquitously expressed CatL when assessing RANKL‐stimulated RAW264.7 cells and BMDMs (Figure S2).

Previous studies have reported how cytosolic CatB can be dramatically activated by intracellular ceramide species of mammalian origin.^23^, ^32^ Thus, we initially examined the affinity of PGDHC to mouse cytosolic CatB using a pulldown assay in which RANKL‐stimulated RAW 264.7 cell lysate was bound to PGDHC‐coupled glass beads. Western blot analysis using a CatB antibody resulted in 38 and 50 kDa bands, corresponding to CatB and pro‐CatB respectively (Figure 2E). These data indicated that PGDHC physically interacts with the cytosolic CatB in OCPs. Subsequently, we examined the effect of PGDHC on the enzymatic activity of mouse recombinant CatB protein (rCatB) *ex vivo*. As expected, PGDHC significantly increased the substrate cleavage by rCatB in a time dependent manner (Figure 2F,G). Furthermore, the increase of rCatB enzymatic activity produced by PGDHC was comparable with that of mammalian long chain ceramides C16:0 and dihydro C16:0 ceramides (Figure S3), demonstrating similar catalytic effects of mammalian and bacterial ceramides on recombinant CatB protein *ex vivo*.

Finally, we assessed the effects of PGDHC on the expression of *CatB* mRNA, as well as its enzymatic activity, *in vivo* using a mouse calvaria osteolysis model. Local calvaria injections of RANKL and RANKL/PGDHC significantly enhanced the expression of *CatB* mRNA in bone tissue lysates compared with the group that received vehicle injections, and this increase was significantly higher in the RANKL/PGDHC rather than in the RANKL only experimental group (Figure 2H). Unexpectedly, the CatB enzymatic activity was only dramatically increased in response to the local injection of a mixture of RANKL/PGDHC and not in the mice injected with RANKL or vehicle alone (Figure 2I,J). In conformity with the *CatB* gene expression observations, local injections of RANKL/PGDHC significantly enhanced the presence of TRAP+osteoclasts in sagittal sections of the mouse calvaria compared with injections of RANKL or vehicle alone (Figure S4). Altogether, these data indicated that *P*. *gingivalis*‐*derived* PGDHC can directly interact with cytoplasmic CatB increasing its enzymatic activity in osteoclasts *in vitro* and *in vivo*.

### 3.3 Loss of CatB abrogated osteoclastogenesis promoted by *Porphyromonas gingivalis*‐derived PGDHC

To establish the role of cytosolic CatB in osteoclastogenesis elevated by *P*. *gingivalis*‐derived ceramides, we investigated the possible effect(s) of CA074‐ME, a cell permeable CatB inhibitor, on RANKL‐primed and PGDHC‐treated RAW264.7 cells (Figure S5A). RANKL‐primed cells treated with PGDHC had significantly higher expression of important genetic markers for osteoclastogenesis, including the nuclear factor kappa‐B (NfkB), nuclear factor of activated t cells 1 (*Nfatc*‐1), osteoclast stimulatory transmembrane protein *(Oc*‐*stamp*), tartrate resistant acid phosphatase *(Acp5*/*Trap*) and cathepsin K (*CatK*) compared with RANKL‐primed and untreated cells (Figure S5B–F). Interestingly, the increased expressions of *NfkB*, *Ocstamp* and *Acp5*/*Trap*, but not *CatK* were abrogated by the addition of CA074‐ME (Figure S5B–F), which indicated that the CA074‐ME selectively inhibited CatB and that CatB mediated the osteoclastogenic effects of PGDHC in RAW267.4 cells. Moreover, PGHDC increased the emergence of TRAP+cells and their demineralizing activity, which were significantly reduced in the presence of CA‐074ME (Figure S5G,H). In contrast, no significant effect was observed on the PGDHC‐enhanced osteoclastogenesis after addition of the CatL inhibitor, CAS‐108005, indicating that PGDHC‐mediated osteoclastogenesis is promoted by intracellular CatB (Figure S6).

**FIGURE 3:**
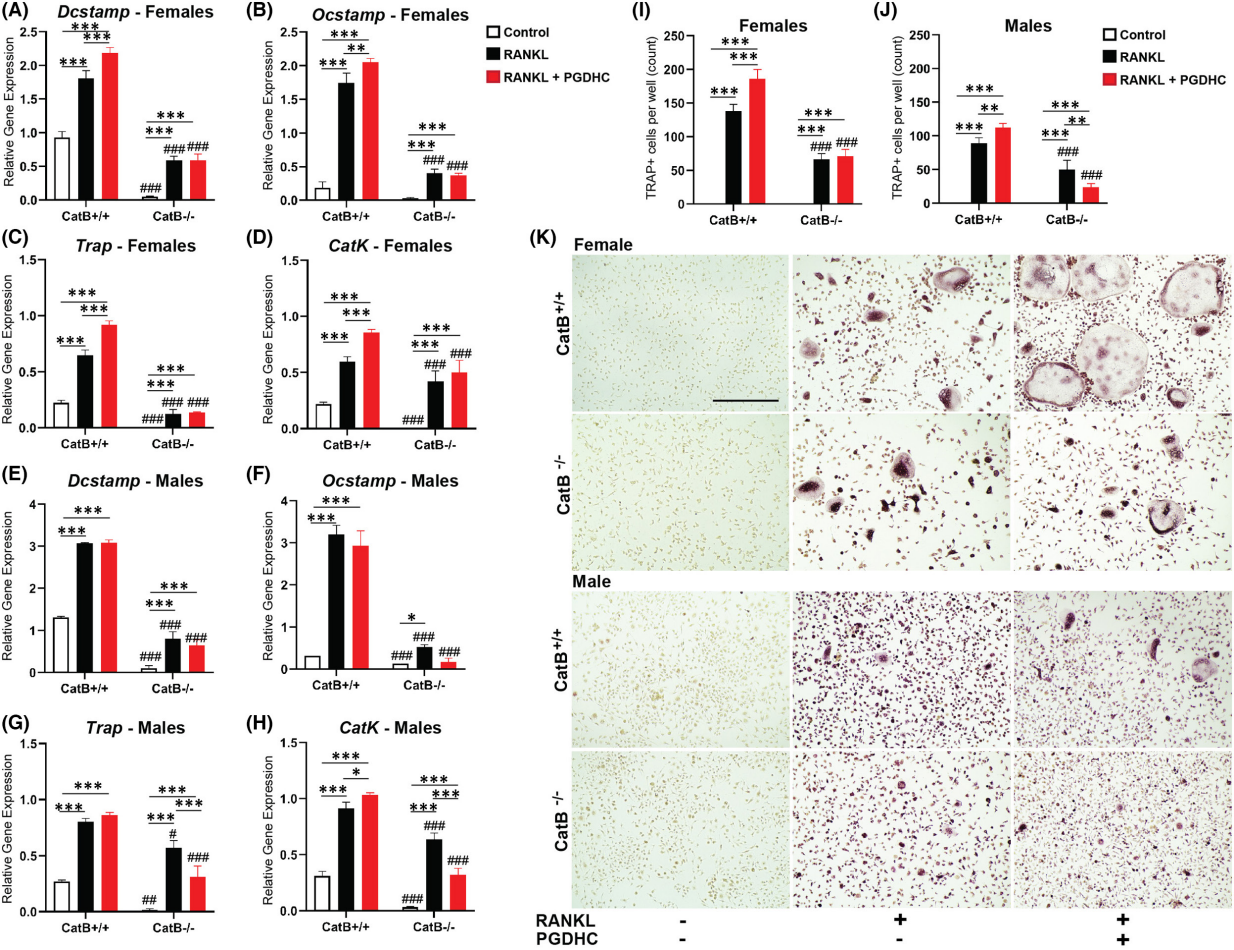
Effects of PGDHC on *in vitro* RANKL‐induced osteoclastogenesis using bone marrow derived macrophages (BMDMs) isolated from wild‐type (CatB^+/+^) and CatB‐*knock out* (CatB^−/−^) mice. Expression patterns of some osteoclast fusion and differentiation mRNA genes in RANKL‐stimulated BMDMs from male and female CatB^+/+^ and CatB^−/−^ mice were measured by qRT‐PCR analysis and are presented as the fold change after normalization against the housekeeping gene GAPDH (A‐H). The number of osteoclasts differentiated from male and female CatB^+/+^ and CatB^−/−^ RANKL‐stimulated BMDMs are presented as the total count of tartrate resistant acid phosphatase positive (TRAP+) multinucleated cells per treated well (I‐J). Representative images of TRAP+multinucleated cells from each experimental group (K). *p < 0.05; **p < 0.01; ***p < 0.001; (#*p *< 0.05; ##*p *< 0.01; ###*p *< 0.001) between CAtB^+/+^ and CatB^−/−^ counterparts; Scale bar (200 μm) applies to all images

In view of these results, we tested next the impact of PGDHC/CatB axis in RANKL‐primed osteoclastogenesis using BMDMs isolated from male and female CatB^+/+^) and CatB^−/−^ mice. To the best of our knowledge, *in vitro* results showed that PGDHC significantly increased the expression patterns of osteoclast‐specific cell fusion and activity markers, including the dendrocyte expressed seven transmembrane protein (*Dc*‐*stamp)*, *Oc*‐*stamp*, *Acp5*/*Trap* and *CatK*, in BMDMs from female CatB^+/+^ (Figure 3A–D) relative to both untreated and RANKL‐primed BMDMs, whereas only *CatK* mRNA expression was increased in RANKL/PGDHC‐treated male CatB^+/+^ BMDMs compared with other treated groups (Figure 3H). Moreover, no or little difference was observed on the expression of all the mentioned osteoclast‐specific cell fusion and activity markers after stimulation with PGDHC in RANKL‐stimulated CatB^−/−^ BMDMs isolated from both male and female mice, compared with RANKL‐only stimulated cells. Finally, the emergence of TRAP+osteoclasts was significantly elevated in RANKL‐stimulated CatB^+/+^ OCPs isolated from male and female mice, with notably larger osteoclasts deriving from female BMDMs, compared with those observed in CatB^−/−^ OCPs. These data demonstrate the advantage in osteoclast differentiation promoted by PGDHC via CatB.

Finally, we evaluated the impact of PGDHC on *in vivo* osteoclast activity in the calvaria of CatB^+/+^ and CatB^−/−^ male and female mice using live mouse imaging and immunohistochemistry assays.^33^ Local injections of RANKL or a combination of PGDHC and RANKL significantly elevated the activity of osteoclasts in a calvaria of male and female CatB^+/+^ mice compared with those mice injected only with the vehicle, with significantly higher activity in mice injected with the combination of RANKL and PGDHC (Figure 3A–D). In contrast, no or little effect of locally injected PGDHC on osteoclast activity was detected in the calvaria of male and female CatB^−/−^ mice. It is noteworthy that the emergence of TRAP+cells at the calvaria lesion was elevated in both male and female CatB^+/+^ mice receiving RANKL/PGDHC compared with CatB^−/−^ mice (Figure 3E–H).

## 4 DISCUSSION

Our understanding of the molecular mechanisms underlying pathogenic bone resorption induced by dysbiosis has been advanced in the last decade by the finding that numerous bacterial lipid‐virulence factors, including LPS and ceramides, are prominently engaged in osteoclastogenesis and inflammation. Various lines of evidence indicate that dysbiosis at the periodontal site is known to result from *P*. *gingivalis* interference with the host immune response leading to alveolar bone loss. While P. gingvalis‐derived LPS is detected in negligible amounts in human periodontitis lesions several novel sphingolipids isolated from *P*. *gingivalis*, including phosphoethanolamine dihydroceramide (PEDHC) and phosphoglycerol dihydroceramide (PGDHC), were identified in substantially greater amounts in inflamed human periodontal tissues when compared with LPS.^12^, ^18^ Although the effect of PGDHC in cells of the oral mucosa has not been widely studied, it can induce the secretion of inflammatory factors by primary gingival fibroblasts.^12^ In the present study, we determined for the first time that phosphoglycerol dihydroceramide (PGDHC) increases RANKL‐induced osteoclatogenesis in a cytosolic cathepsin B (CatB)‐dependent manner. Our studies also showed that PGDHC promotes lysosomal membrane permeabilization (LMP) and enhances the expression and activityof CatB in mouse osteoclast precursors (OCPs) and, consequently, increases RANKL‐primed osteoclastogenesis *in vitro* and *in vivo*. Moreover, the loss of CatB abrogated the osteoclastogenic enhancement mediated by PGDHC.

Cathepsin B is a lysosomal protease released into the cytosol following LMP,^34^ a mechanism of cellular homeostasis involving lysosomal and recruited proteins, including galectin 3 (LGALS3) and lysosome associated membrane protein 1 (LAMP1).^35^ LGALS3 is a member of the lectin family involved in lysosomal repair^36^that is considered a marker of lysosomal and endosomal damage.^37^ LGALS3 has high affinity for cells expressing LAMP‐1,^38^ which is known to stabilize the lysosomal membrane after LMP.^30^, ^31^ The identification of both proteins during RANKL‐induced osteoclastogenesis and their dysregulation by PGDHC confirms the lysosomal leakage and disruption triggered by PGDHC in OCPs (Figure 1). In addition, LGALS3 is highly expressed in areas of bone resorption but its expression declines during osteoclastogenic differentiation,^39^, ^40^ as LGALS3 can disrupt early osteoclastogenic gene expression and hinder osteoclastogenesis.^39^ Similarly, LAMP1 overexpression is associated with inhibition of osteoclastogenic activity^41^ and decreased traffic of lysosomal enzymes, including CatB and CatD, into the cytosol.^30^ Our data show that PGDHC inhibits the expression and co‐localization of Lgals3 and Lamp1, which may favor CatB leakage into the cytosol, as well as osteoclastogenic gene expression and differentiation.

**FIGURE 4:**
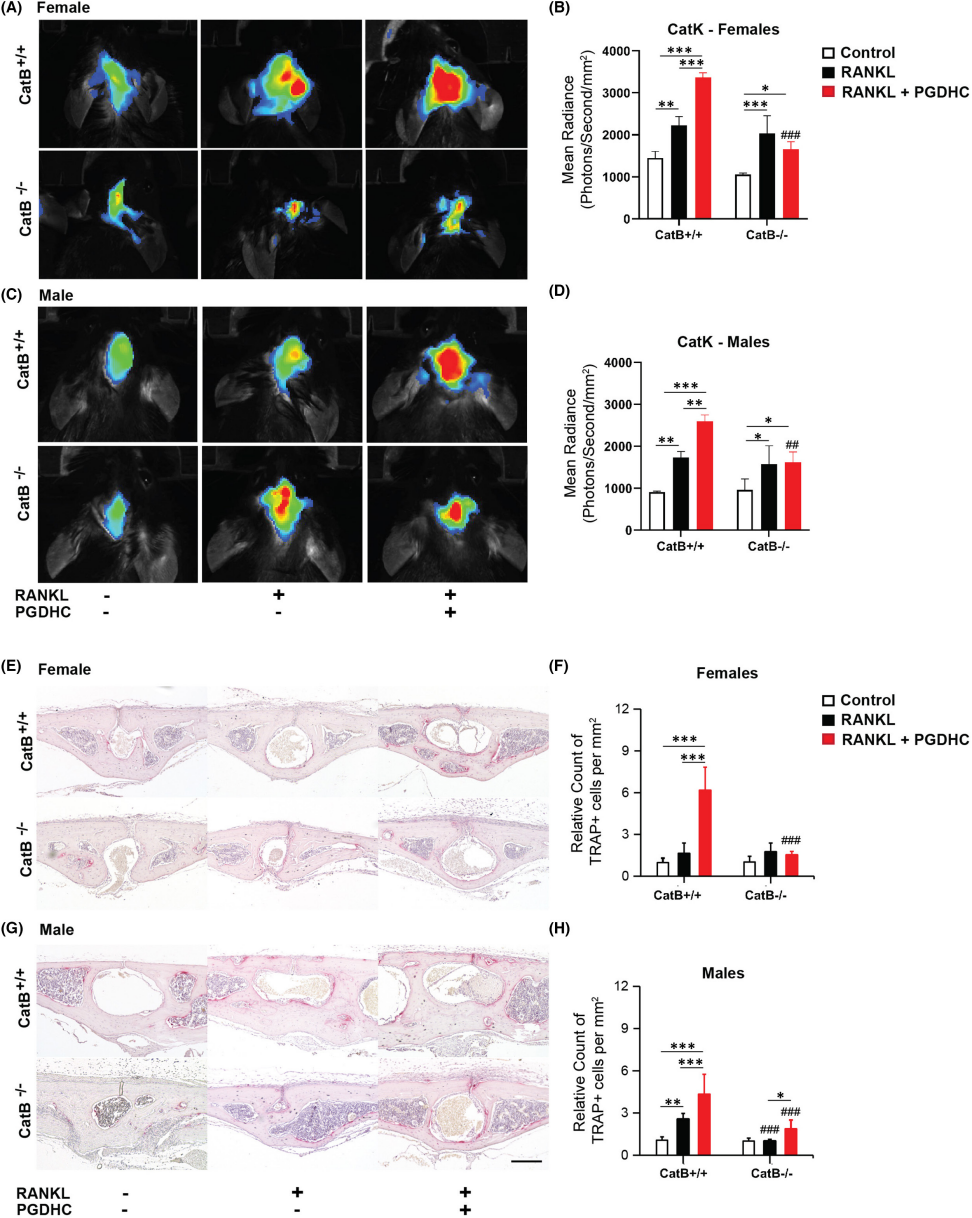
No or limited effect of PGDHC on RANKL‐mediated osteoclastogenesis induced in CatB^−/−^ mouse calvaria *in vivo*. (A, C) Live imaging and (B, D) quantification of male and female wild‐type (CatB^+/+^) and CatB knock‐out (CatB^−/−^) mice calvarias injected with RANKL or a combination of RANKL‐PGDHC, using *in vivo* bioluminescence imaging (Extreme II, Bruker) with a CatK specific fluorescent probe (Pro Sense 680, Perkin Elmer). (E, G) Histological staining of TRAP+cells on the sagittal suture/parietal bones of the experimental subjects detailed above. Haematoxylin was used as a nuclear counterstain. (F, H) The normalized number of TRAP+cells per mm^2^ represent the increase in TRAP positive cells identified in the male and female wild‐type (CatB^+/+^) and CatB knockout (CatB^−/−^) mice calvarias injected with RANKL or a combination of RANKL‐PGDHC

Ceramides are bioactive sphingolipids involved in cell apoptosis, senescence, and autophagy^8^ that effectively activate CatB.^23^ Long chain ceramides and purified proteases produced by *P gingivalis*, which may include PGDHC, can considerably activate CatB.^23^, ^32^, ^42^ We hereby demonstrate the direct activation of CatB by PGDHC (Figure 2). CatB is temporally upregulated in preosteoclasts and is required for the degradation of non‐muscle myosin IIA (Myh9), an actin binding protein that mediates OCP fusion, adhesion and migration.^19^, ^43^ Furthermore, reduced osteoclastogenesis as a result of CatB inhibition *in vitro* and *in vivo* have previously been reported.^19^, ^44^ Our results confirm these reports, as decreased osteoclast differentiation was observed in CatB^−/−^ mice compared with wild type mice. Previous reports have also determined that *P*. *gingivalis*‐derived PGDHC can increase osteoclast differentiation and the expression of osteoclastogenic markers *in vitro* and *in vivo*.^12^, ^18^ However, the mechanisms by which PGDHC enhances RANKL‐induced osteoclastogenesis were unclear. The data collected in this study confirm the increased osteoclastogenesis due to PGDHC stimulation but, more importantly, we demonstrate that the osteoclastogenic effect of PGDHC is not observed in the absence of CatB (Figures 3 and 4). The deletion of CatB in CatB^−/−^ mice resulted in complete abrogation of PGDHC induced osteoclastogenesis, which demonstrates that the osteoclastogenic effect of PGDHC is dependent on CatB and its enzymatic activation.

In addition to our observations on the effect of the PGDHC/CatB axis on osteoclastogenesis, we have observed a greater osteoclastogenic effect in female rather than male wild type mice throughout our *in vitro* and *in vivo* studies. In contrast, PGDHC’s osteoclastogenic effect was equally abrogated in both male and female CatB^−/−^ mice (Figures 3 and 4). The intrinsic differences between male and female immune cells, which include monocytes that can differentiate into osteoclasts, have been reported in chicken and mice.^45^, ^46^ In fact, these sex‐related dysmorphisms may account for increased immune responses and immunogenic gene expression in females.^45^, ^46^

This study has potential limitations including additional physiological factors that may have affected the expression of cathepsins *in vivo* after calvaria injections, as well as the existence of compensatory processes as a result of the absence of CatB in our CatB^−/−^ mice, which may be sex dependent. The mechanisms by which PGDHC affected lysosomal homeostasis and by which sex dysmorphisms affected osteoclast differentiation where not extensively examined nor discussed since they fell outside of the scope of this study.

Altogether, these results are in agreement with previous reports of reduced osteoclastogenesis as a result of CatB inhibition *in vitro* and *in vivo*.^19^, ^44^ Furthermore, our results suggest that PGDHC isolated from *P*. *gingivalis* can enhance osteoclastogenesis *in vivo* via CatB‐dependent signaling. These findings indicate that the novel class of bioactive sphingolipids produced by the key periodontal pathogen P. gingvalis has the potential to elevate osteoclastogenesis via interaction with lysosomal CatB in OCPs. We posit that our studies provide a strong rationale that non‐eukaryotic ceramides remain to be further explored for their roles in inflammatory osteolysis.

## CONFLICT OF INTEREST

The authors have no conflicts of interest to declare.

## AUTHOR CONTRIBUTIONS

**Carolina Duarte:** Data curation (equal); Formal analysis (equal); Methodology (equal); Writing – original draft (equal). **Chiaki Yamada:** Formal analysis (equal); Investigation (equal); Methodology (equal); Validation (equal). **Christopher Garcia:** Investigation (equal); Visualization (equal); Writing – original draft (equal). **Juliet Akkaoui:** Formal analysis (equal); Investigation (equal); Validation (equal). **Anny Ho:** Formal analysis (equal); Validation (equal). **Frank Nichols:** Formal analysis (equal); Funding acquisition (equal); Methodology (equal); Resources (equal). **Alexandru Movila:** Conceptualization (lead); Formal analysis (lead); Funding acquisition (lead); Methodology (lead); Project administration (lead); Writing – original draft (lead).

## ETHICS APPROVAL

All protocols used in animal experiments were approved by the Animal Care and Use Committee at Nova Southeastern University (IACUC approval #2019.01.AM3‐A3) in compliance with the guidelines set forth by National Institutes of Health.

## Supporting information

Fig S1‐S6
