# Supplementary figures and images for: Crosstalk between dihydroceramides produced by Porphyromonas gingivalis and host lysosomal cathepsin B in the promotion of osteoclastogenesis
Source: J Cell Mol Med. 2022 Apr 16;26(10):2841–51. doi: 10.1111/jcmm.17299 (PMC9097840; doi:10.1111/jcmm.17299)

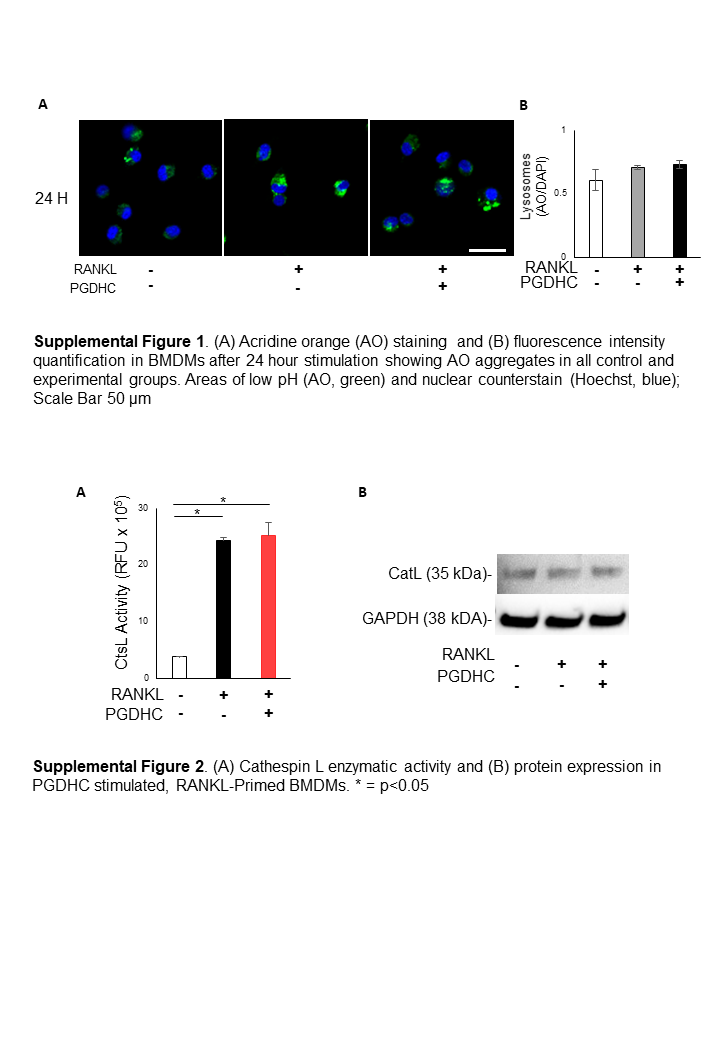

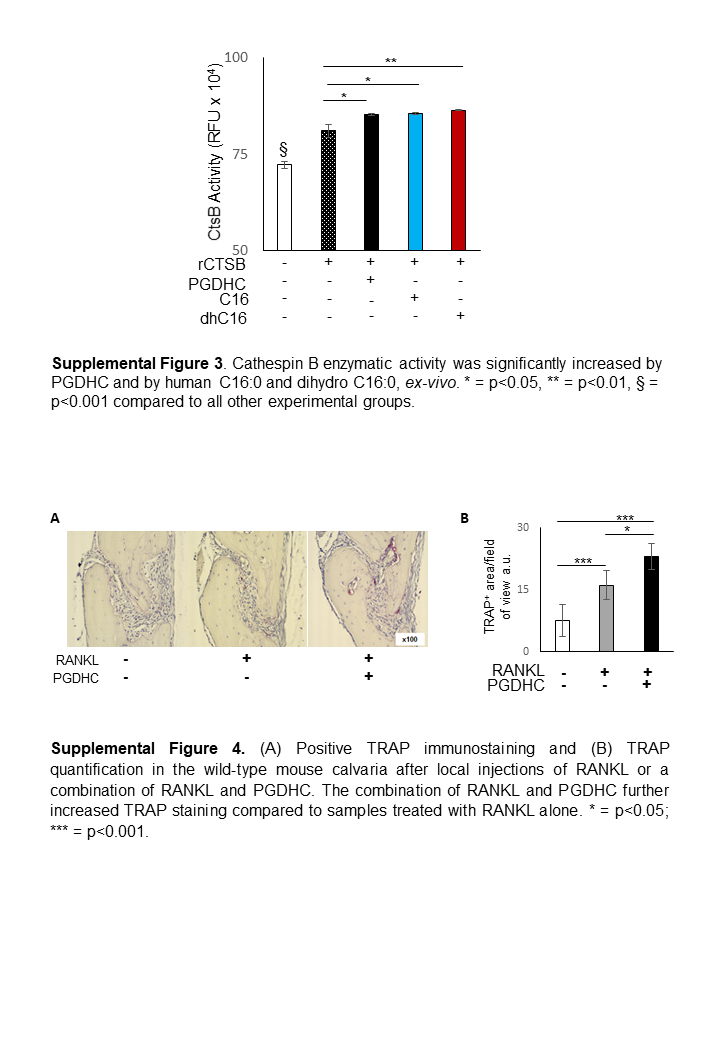

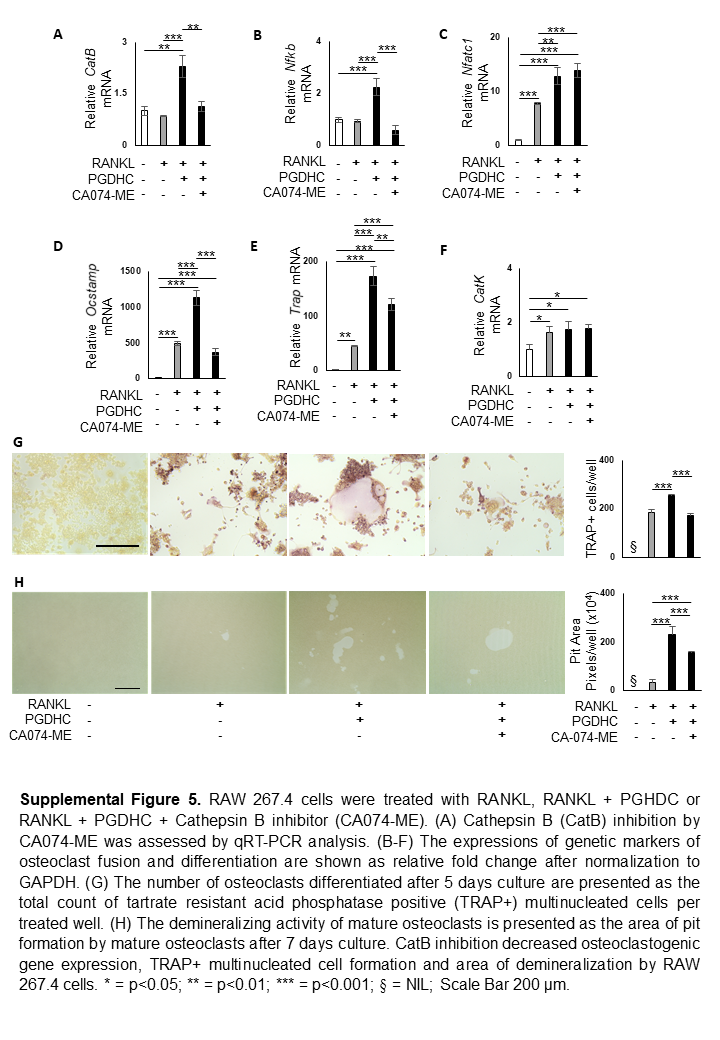


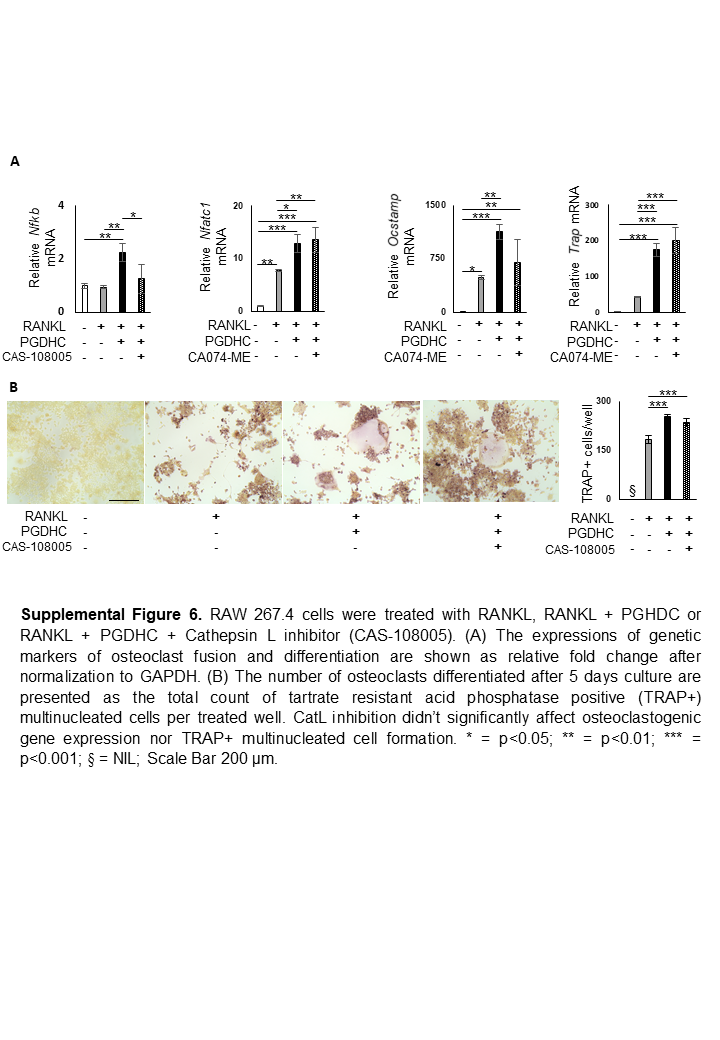

Supplement: Supplementary file 1 — Fig S1‐S6 [file JCMM-26-2841-s001.docx]
